# Number without language: comparative psychology and the evolution of numerical cognition

**DOI:** 10.3389/fpsyg.2013.00295

**Published:** 2013-05-23

**Authors:** Christian Agrillo, Michael J. Beran

**Affiliations:** ^1^Department of General Psychology, University of PadovaPadova, Italy; ^2^Language Research Center, Georgia State UniversityAtlanta, GA, USA

Curiosity about the numerical abilities of non-human animals has been a topic in experimental psychology for about as long as such a science has existed. Some of the earliest comparative work looked at how well animals could use number as a cue in making different kinds of judgments. Early results were mixed, and in some cases were incredibly controversial (e.g., the case of the horse Clever Hans). After the “fall” of behaviorism and the “rise” of cognitive psychology, the question of numerical competence became one of the dominant research areas in the new field of comparative cognition. The present series of papers in this special topic represents the newest additions to that research area. This special topic also serves as an anniversary of sorts, as it comes 25 years after one of the most influential papers on the subject of numerical competence in animals (Davis and Perusse, 1988). Comparing the major themes of that paper to the topics in this special issue serves to highlight where we came from, and where we might be going with future research.

Davis and Perusse (1988) and the commentaries that were involved in that paper presented a number of critical issues in the area of numerical cognition research at that time. One of the largest was whether the use of the term “counting” was appropriate for much of what was being studied with non-human animals, and they cautioned against applying that term to methodologies that did not require the principles that underlie counting in humans (e.g., Gelman and Gallistel, 1978). This problem seems to be largely resolved, as most researchers in the area today recognize that animals are not counting, even in their most sophisticated demonstrations. Davis and Perusse (1988) attempted to standardize terminology. Some of the terms they offered have come to be re-defined (e.g., “sense of number”) whereas others have come to be re-named (e.g., changing *relative numerousness judgments* to *relative quantity judgments* where it is clear that number is not the only stimulus dimension that an animal could use to perform a task). Subitizing remains an interesting phenomenon, sometimes evident in animal research but other times not evident (see Murofushi, 1997). Davis and Perusse (1988) also called for the use of transfer tests, and better controls in experimental work, and although those concerns still remain (e.g., Beran, 2012), in general the field has risen to that challenge. Perhaps the biggest change has been the shift from questioning whether number is used by animals only as a “last resort” (e.g., Davis and Memmott, 1982) to the now general consensus that number is a relevant stimulus cue to which animals are sensitive in a number of contexts (e.g., Cantlon and Brannon, 2007; Agrillo et al., 2011). As the papers in this special topic demonstrate, animals can and do use quantitative and numerical information in a variety of contexts, and in some cases may even be fairly described as numerate, although with certain caveats such as being far more “fuzzy” in how they represent numerosities than are humans above 5 or 6 years of age.

This special topic encompasses 16 novel studies, including mammals (e.g., Beran et al., 2012; Panteleeva et al., 2013), birds (Armstrong et al., 2012), fish (Gómez-Laplaza and Gerlai, 2012), and invertebrates (Pahl et al., 2013). We learned that numerical information is a potential relevant cue also in species that have been poorly investigated compared to primates (e.g., dolphins: Yaman et al., 2012, beetles: Carazo et al., 2012). Some non-numerical visual cues, however, may play a key role too: for instance, overall quantity of movement within the shoals is a necessary condition for large (but not for small) shoals discrimination in angelfish, with interesting implications for the theoretical debate about how non-human animals process small and large quantities (Gómez-Laplaza and Gerlai, 2012). Also the lack of a ratio effect reported in wolves in the range 1–4 is potentially in line with the idea of different ways to process small and large quantities (Utrata et al., 2012). In contrast, data against the existence of a subitizing-like process have been described in primates (Jones and Brannon, 2012; Barnard et al., 2013) and canids (Baker et al., 2012), highlighting that we are still far from solving the question raised by Davis and Perusse regarding whether animals subitize (see also Cutini and Bonato, 2012). Interspecific studies comparing different species (fish: Agrillo et al., 2012; canids: Baker et al., 2012) are also reported in the special topic: in both of these studies similarities among the species are greater than differences, suggesting the existence of similar numerical systems among vertebrates.

The special topic also includes theoretical and research studies on human infants. In particular, attention has been focused on number-space interaction (de Hevia et al., 2012), the relation between numerical and non-numerical cues (Uller et al., 2013) and the development of ordinal abilities (Anderson and Cordes, 2013). It has been suggested that human and non-human animals share the same non-symbolic numerical systems (Feigenson et al., 2004). If so, we believe that an inter-disciplinary approach including cognitive (non-verbal numerical judgments in adults), developmental (newborns and infants) and comparative psychology will represent the very frontier of numerical cognition studies, enabling us to understand both the evolutionary foundations of our numerical abilities and the exact mechanisms underlying quantity discrimination in the absence of language.
